# Fears towards disability and their impact on teaching practices in inclusive classrooms: An empirical study with teachers in Greece

**DOI:** 10.1016/j.heliyon.2023.e16332

**Published:** 2023-05-16

**Authors:** Olga Lyra, Kyriaki Koullapi, Eirini Kalogeropoulou

**Affiliations:** a Inclusive Education, School of Education and Social Sciences, Frederick University, Nicosia, Cyprus; b School of Education and Social Sciences, Frederick University, Nicosia, Cyprus; c School of Education, Open University of Cyprus, Nicosia, Cyprus

**Keywords:** Fears, Teachers, Resistance to change, Culture of disability, Inclusive education, Mental models

## Abstract

Our research examines Greek special and general education teachers' fears toward disability and their impact on teaching in inclusive classrooms. We interviewed 12 teachers from the region of Attica (Athens) and documented attitudes and beliefs toward disability, with the goal of identifying teachers' personal sources of resistance to inclusion. Medical paradigm of understanding disability and the absence of inclusive school culture are some of the results that revealed teachers’ sources of resisting to inclusive changes and the way these affect their teaching. Based on these findings, we discuss a two-pronged process that shifts the existing culture of understanding disability and welcomes diversity in schools.

## 1 Introduction

### 1.1 Background

During the past decades there has been a worldwide effort to provide equal educational opportunities to all students [1]. A significant effort has been put on applying the principles of inclusive education in mainstream schools, following the Salamanca Statement [2], the United Nations Declaration on the Rights of Persons with Disabilities [3] as well as the Agenda 2030 and The Sustainable Development Goals [1]. However, at the 56th session of the United Nations Commission on Social Development it was ascertained that several countries, Greece among them, so far have failed – to a certain extent – to meet these goals [4].

Based on Ainscow's understanding (2010), inclusive education is an ongoing and evolving process in which the educational system seeks to respond to the specific needs and learning profiles of all students, through the necessary adjustments and changes, both, in respect to the curriculum and to the infrastructure, the practices, the policies and the school culture.

According to the European Agency for Special Needs and Inclusive [5] and research findings, e.g. Refs. [6,7]; if teachers aren't in a state of readiness for applying the values related to inclusive education, such as social justice, ethics of education [8] and ethics of care [9], the efforts made through policy-making for the implementation of inclusive education will remain one-sided and, thus, will not be effective enough. Research has shown that teachers' hesitation to teach in classrooms of mixed abilities is related to their views on inclusive education [10], as well as on their perceived fears regarding the challenges in a mixed-ability school environment. In fact, according to Senge's concept of schools as evolving learning organizations (2006), fear is a considerable part in teachers' mental models and, consequently, an obstacle in teachers' behavior.

The above remarks are associated to the theory of behaviorism since, according to John Watson, people learn to feel fear, almost in the same way they learn everything else in their lives, and this applies to irrational fears as well. That form of fear mobilizes the behavior of avoidance and guides to a state of relief and security [11]. According to Ref. [12]; endogenous addictive stimuli, such as thought or mental images, can also create fear. Thus, teachers’ fears toward disability are not only due to issues related to environmental, or other external factors, but also from deeper personal sources.

It is apparent that, although many teachers express positive views towards inclusion, however, the fear and uncertainty of teaching students with disabilities prevails due to several aspects ([81]; [13]. For example, stereotypes, negative perceptions, as well as the prevalence of the charitable model of understanding disability, awaken feelings of fear towards the prospect of inclusion and in particular towards disability. Further, ineffective cooperation between general and special education teachers and the lack of sufficient knowledge are some deterrent aspects [7,14]). Previous research, also suggests that their fears towards disability are associated with the lack of appropriate knowledge and practice [7], the unfamiliarity with disability, as well as with prejudices and stereotypes, some of which go back to their experiences at young age [15].

### 1.2 Objectives

With our study we aim at shedding light to the fears towards disability that general and special education teachers express, since they hold responsibility for supporting learning for all students in classrooms of mixed abilities. Especially, we aim at investigating, whether the factors discussed above prevent teachers in Greece from overcoming such personal fears, applying inclusive practices in their daily teaching and embracing inclusive school culture overcome such personal fears, even after twenty-two years of the first organized educational legislation for inclusion in Greece (Law 2817/2000).

## 2 Literature review

### 2.1 Inclusive education

UNESCO (2008), in an attempt to help educational systems worldwide to effectively adopt in practice the values of full inclusive education, describes the key factors of inclusive education for all students: (a) promoting student participation and reducing exclusion from and for education; (b) the presence, participation and achievement of all students, but especially of those who are excluded or at risk of marginalization. Following these principals, becomes clearly understood that in the context of inclusive education is important the adoption of “a socio-ecological approach regarding the interactions between students’ capabilities and environmental demands, stressing that educational systems must adapt to and reach all students – and not vice versa- ([78], p. 2).

Inclusive education has been shown to have positive effects in terms of learning and social participation for all students, regardless of different gender, abilities, ethnic, linguistic and socio-economic background, migration biography etc. [16,17]. Research shows that, in order for inclusive education to be successfully implemented, certain conditions must be met [6,18,19]. In particular, the shift of school culture towards inclusiveness is of major importance, as this has been shown to be the cornerstone for successful change, since inclusive culture aims at encouraging all school community members to embrace diversity through collaboration [[20], [21], [22], [23]]. For all this to succeed, teachers need to manifest the appropriate attitude.

### 2.2 Teachers’ attitudes and beliefs towards inclusive education and the impact of fear

A teacher's response to implementing change is rooted in her/his belief system evolving from experiences and personal values [24] that are internally and externally assimilated [25]. Attitudes, on the other hand, develop out of ongoing thought processes that are embedded in beliefs [26,27], encompassing many mental constructs such as views, conceptions, dispositions, that an individual has about an object, person or group [28].

Their negative beliefs about disability are likely to be source of internal fears about the prospect of inclusion. As [29] states, a person's resorting to avoidance in order not to feel fear, is a reinforcing factor to repeat the avoidance behavior when he experiences the addictive phobic stimulus again, so his phobia is maintained. Signals of fear might be emerged due to neutral stimuli that, in a particular situation, are of relevance and the repetition of the connection between fear and the new phobic stimuli is going to intensify the fear [30].

Rachman's theory of fear acquisition supports that, it is probable that informational and instructional processes provide the basis for most of our commonly encountered fears of everyday life [79]. According to Ref. [31]; many of the fears we experience are linked to the culture of fear. As he puts it (p.5) “the free-floating dynamic of fear is promoted by a culture that communicates hesitancy and anxiety towards uncertainty and continually anticipates the worse possible outcome”. As a consequence, individuals prefer conditions that offer them safety. Thus, the culture of fear directs people to the avoidance of taking any kind of risks in their life because it is a form of culture that promotes precaution as a virtue and cultivates a climate, where taking risks is even considered irresponsible behavior.

As to teachers' attitudes, they appear to be of the most prominent factor for the implementation of inclusion. The term ‘attitude’ is generally conceived as an evaluation of knowledge or feelings, which refers to any concept people may hold in their mind, concrete or abstract [19]. Among the most common factors that appear to have an influence on teachers' attitudes are the following: gender [32]; [80], educational background and additional specialized training [33,34], working conditions and school environment [35]) and previous experience with students with disability [35]). At the same time, prejudices and stereotypes are also considered factors that affect teachers' attitudes towards inclusion [36].

[19] pointed out that, even though teachers may have positive attitudes toward inclusive education, some do not agree on “total inclusion” and that attitudes are strongly influenced by educational environment-related variables (e.g., adequate infrastructure, availability of human support) and child-related variables (e.g., the nature of students' disabilities), something that is directly influenced by the medical paradigm of understanding disability [6] Within the medical paradigm, disability is seen to arise as a direct consequence of a person's biological make-up and functioning, something that lead teachers who embrace this way of understanding disability, to develop negative attitudes toward the prospect of “total inclusion” [17]. Positive attitudes can lead to higher expectations in respect to learning outcomes and social acceptance, thus increasing learning success for all students [36,37].

Teachers' attitudes and fears are related to one of the five disciplines suggested in Senge's theory of organizational learning, according to which each school must be acting as learning organization, i.e., “continually expanding its capacity to create its future” [38]; p.14). In order for a school to achieve this goal, he identifies five disciplines that must be followed: “systems thinking”, “personal mastery”, “mental models”, “shared vision” and “team learning” [[38], [39], [40]].

When that happens, teachers become more engaged in helping to create a successful environment for students and themselves [38,39]. As to the third discipline, “mental models”, they are the maps that individuals and organizations follow to help themselves to make sense of their context or world and to interpret their reality [40]. According to Ref. [38]; attitudes and beliefs are factors that are associated with one's working mental models. He argues that individuals develop strategies to cope with facts, data, events etc. that are unfamiliar, or oppose to what they know and operate upon. In fact, they develop internal resistance to change that refers to internal factors and inner motivation. In the case of teachers, this refers to their mental models behind their reaction to inclusive education [38,39].

Teachers' resistance to innovation in schools and ways to increase their motivation and engagement in change efforts are frequently asked research questions. According to Ref. [41]; the term resistance “is commonly used with a negative connotation to indicate an oppositional action to something that one disapproves” (p. 839). The sources of resistance are usually connected with someone's believes, culture, or even fear for something he/she is unacquainted with [38,41]. As [38] points out, individuals are not from the beginning reluctant to external changes, but to changing themselves.

Research findings indicate that teachers' fear might be manifested with the avoidance of teaching under particular circumstances, i.e., classrooms of mixed abilities [14]; [42]. The existence of teachers' fear toward inclusion is possible to be connected to the lack of confidence in their teaching ability, unwillingness for collaborative teaching, lack of adequate teaching methods, inability to engage their students or to their insufficient knowledge about inclusive practices, since other researches investigating teachers’ fears in different circumstances have concluded to these elements [15,42].

As to inclusion, the above is related to teachers' perception about their role in teaching in inclusive settings. They are reluctant to change their daily routine as this requires more preparation at home and familiarity with new teaching methods [43]. They state that they do not have sufficient knowledge and training to meet the requirements of mixed ability classes, so they appear to prefer the status quo [[43], [44], [45]]. Sometimes this kind of reluctance might indicate feelings of ‘ownership’ of the classroom, thus making collaborative teaching more challenging [46]. This attitude is not in line with the concept of ‘ethics of education’ [47] and ‘ethics of care’ [9], which are important for the development of an inclusive school. Since fears, stereotypes and lack of culture for inclusive education prevent teachers from fully embracing the responsibility for fair quality education for all students [48]. After all, as [48,49] explain, ‘ethics of care’ is a matter of caring, involvement and maintenance of harmonious relationships, which underpin education ethics and thus, both of them support and enhance inclusion.

With this research, we believe is important to investigate, whether the factors discussed above prevent teachers in Greece from overcoming such personal fears, applying inclusive practices in their daily teaching and embracing inclusive school culture overcome such personal fears, even after twenty-two years of the first organized educational legislation for inclusion in Greece (Law 2817/2000).

### 2.3 The case of Greece

Greece is a state member of the European Union since 1981. In the last years Greece has faced significant challenges in societal and economic level on the one hand due to the severe economic recession and the high unemployment rates, and on the other hand due to the migration flow that contributed to a rapid change in the country's population, which consequently sets new challenges for the schools. For those reasons Greek schools face the challenge to adapt to this new reality and especially to the broad diversity of the students, in terms of abilities, religions, socio-economical, ethnic and linguistic background, thus preparing new citizens to appreciate, respect and accept differences among people. Therefore, schools need to disengage themselves from their traditional role and teaching methods and become agents of change [38].

In accordance with the guidelines of international conventions for the implementation of inclusive education policies ([3]; UNESCO, 2009), the current Greek legislation with the Law 3699/2008 (Ministry of Education, Lifelong Learning and Religious Affairs, 2008) places an emphasis on supporting inclusion, with the aim of ensuring obligatory state education and equal opportunities for all students at all school levels. In fact, inclusion is organized in various arrangements within the mainstream school, i.e. in regular classrooms without additional support besides the classroom teacher; in regular classrooms with in-class support provided by a special education teacher; finally, in ‘integration classes’ within the mainstream school. The latter type is the most common model of support for students with disabilities [50]. ‘Integration classes’ are according to Ref. [51] similar to pull-out programs in US or like the part time withdrawal in a support class of the UK. At the same time special schools still operate mainly for students with severe disabilities.

Efforts by the Greek state to apply the principles of inclusive education sets the challenge for teachers to adapt to new circumstances in a classroom environment that is diverse, as never before. [80] research found that the kind and the severity of the disability led teachers to develop negative attitudes regarding inclusion. In addition, they found that gender, age, adequate training (or the lack of it, reversely), work related stress, the fear of change, and previous experience with students with disabilities also affect teachers' attitude. Further, in their research, [45]; found that the lack of time for preparation, the curriculum and the co-operation between classroom teachers and co-teachers are also factors that affect Greek teachers' attitudes towards inclusion. Similar are the results of Lenakaki et al. (2018) research, who suggest that experience, training, teachers' practices, their fear for teaching successfully students with disabilities and, finally, their prejudice for these students' ability to learn are factors that determine their attitudes towards inclusion. Although these factors have been extensively investigated, they don't seem to adequately explain the sources of teachers' resistance to changes of inclusion. Thus, more in-depth study is needed, regarding the existing school-culture and resources obstructing the establishment of an inclusive culture.

It becomes apparent that it is highly important to investigate the factors -such as culture-that are considered as sources of internal resistance and affect primary school teachers’ attitudes [13], even so because a literature gap is identified in respect to those sources that affect their views towards inclusion [52]. In addition, we found limited qualitative research on this topic, as far as the Greek educational landscape is concerned.

## 3 Methodology

### 3.1 Research purpose and questions

As a result from all the above, through semi-structured interviews, we aim at discussing teachers' beliefs and attitudes in respect to disability and their views as to the feasibility of inclusive practices in their daily teaching. In addition, we aim at investigating how teachers define their role and, consequently, their responsibilities within an inclusive classroom. Finally, we aim at identifying teachers’ fears that act as personal sources of resistance to change, as suggested by inclusive education, and their impact on their practices in the classroom. In particular, our study addresses the following research questions:

1. What are the guiding beliefs and attitudes of special and general education teachers in respect to disability, as well as to the feasibility of inclusive practices in their daily teaching?
2. How do teachers define their role and, consequently, the responsibilities that they undertake in an inclusive classroom?
3. Which factors are identified as inner sources of resistance to change, as it is suggested in inclusive education?

### 3.2 Research method and participants

To answer these questions, we relied on the qualitative approach. Particularly, the content analysis method was used, since the research design was based on our intention to examine the above research questions in depth [53]. We collected our data through purposive sampling, since it was the most appropriate method to ensure that the participant would be appropriate for the purposes of our research [53]. The inclusion criteria for our sample were specific: we aimed at teachers of general and special education, working in general education schools with inclusive settings. Thus, the research sample was composed of seven special education teachers (Table 1) and eight general education teachers (Table 2) from several public primary schools of Attica region, a geographical space in central Greece, where Athens (the capital of Greece) is located. According to Ref. [53]; the sample was enough for saturation. We decided to interview both special and general education teachers, as we aimed at studying comparatively their opinions regarding their roles, training and views on issues related to students with disabilities, since both teach in the mainstream public school. The results apply to this particular research sample and cannot be generalized. As for the ethical consideration of the study, before the interviews, we explained to the headmaster of each school the topic of our research and ask for his permission to interview the teachers. After that, we informed teachers about the necessary details of our research, as for their voluntary participation. We explained that, in case they wanted to interrupt the procedure they should feel free to do it and **signed consent was obtained from all participants for the procedure of the research.** Additionally, we ask their permission to record our conversation. **The study was approved by the Research Ethics Committee of Frederick University of Cyprus**.

**Table 1:** Special education teachers.

Participant	Age	Professional experience (y.)	Teaching in inclusive class (y.)	Training in Special Education (henceforth SP)	Region of Attica
1	39	16	6	PhD in SP	East
2	58	35	12	Seminars/training	North
3	35	13	5	Seminars/training	Southern
4	50	27	11	PhD in SP	West
5	28	4	4	Faculty of SP	East
6	30	9	7	Degree in SP	Southern
7	50	28	5	Seminars/training	North

**Table 2:** **–** General education teachers.

Participant	Age	Professional experience (y.)	Teaching in inclusive class (y.)	Training in special education (SP)	Region of Attica
8	51	28	Partly	Primer knowledge in SP	East
9	56	32	4	Informative seminars/training in SP	North
10	40	16	Partly	Primer knowledge in SP	East
11	40	17	Partly	Informative seminars/training in SP	East
12	53	29	Partly	Primer knowledge in SP	Southern
13	50	25	Partly	Primer knowledge in SP	West
14	55	23	Partly	Primer knowledge in SP	West
15	40	15	Partly	Primer knowledge in SP	Southern

### 3.3 Research design and instrumentation

In order to collect the data, a two-part interview protocol with semi-structured questions was created. Part 1 was dedicated to gathering information related to teachers' views on disability, whereas Part 2 was focused on teachers' opinions regarding inclusive education and their views on their teaching role in inclusive settings. The interview didn't focus on problems, such as the lack of infrastructure, but rather on inquiring teachers' inner sources of resistance to change. The questionnaire contained semi-structured questions that derived from the international validated questionnaires *The Sentiments Attitudes and Concerns about Inclusive Education Scale* (SACIE & SACIE-R) [54,55] and *The Teacher Efficacy for Inclusive Practices* (TEIP) [56]. Some of the questions were modified by the researchers (turning them from closed to open), according to educational research practices and after cross translation [53]. The researchers conducted a pilot interview, in order first to check the extent to which the questions were understood, and thus considered as adequate and, second, to determine the duration of the interview that was estimated at around 30 min. Prior to the questions, the participants were asked regarding their age, training, internships, years of professional experience in general or special education school settings, and location of the school that they were working at the time of the interview, in the broader area of Athens. Also, all ethical aspects were taken into consideration. Particularly, the participants were asked to choose the time and place they preferred for the interview to take place, a consent form was given to them with information on the research and its purposes, they were assured for the anonymity of their responses and they were offered transcripts of the interview to view their responses. Also, because their participation in the research was voluntary, we explained to them that they could withdraw from it whenever they wanted.

### 3.4 Data analysis

Following the approach of content analysis [53] the data were transcribed, recursively read, coded and categorized in themes. This process resulted, in the first round, in 230 initial codes, which gradually turned into 80 codes. The data of the two groups of participants were examined separately, to allow for comparisons. In a second round, a third individual, who was not involved in the research, was asked to repeat the analysis independently, assigning codes to the interview transcript, with the aim of controlling credibility and validity [53]. The codes from the two rounds of analysis were then compared and resulted in the final coding scheme. Table (3) below shows a sample of the comparison of coding results of the two coders.

**Table 3:** Sample of the comparison of coding results of the two coders.

Thematic category	Quotation	Coder 1	Coder 2
Lack of infrastructure and of differentiated learning material.	1. “We don't even have a ramp outside the integration class.?” [I.5]. 2. “did you see our classes? They are not suitable for all students” [I.1]. 3. “for inclusion to be implemented, first of all basic infrastructure, such as elevators should be at every school” [11] 4. “Books have no differentiated exercises” [I.9].	1. Not enough ramps for access 2. Inappropriate classes 3. No elevators 4. No differentiated materials	1. lack of ramps 2. problematic classrooms 3. “All schools should have elevators” 4. lack of differentiated learning materials
Feasibility of the implementation of the ‘full-inclusion’ model	1. “This student enters the class, but he doesn't earn much” [I.2]. 2. “Special Education Needs (hencefsorth SEN) students cannot attend and keep up with the rest of the general class” [I.8] 3. “Can a SEN student learn to count until 100 by the end of the first class of primary school?” [I.10]; 4. “also it's rarer for him compared to his classmates of typical development to make friendships” [I.2].	1. no benefits from the general class 2. cannot attend the general class 3. doubts 4. no friends	1. like a visitor in the class 2. cannot cope with the general classroom 3. doubts for the effectiveness 4. friendship problems
Teachers' definition of their role and of their responsibilities in an inclusive classroom	1. “We cannot collaborate. I mean I am not sure if that can work” [I.14]. 2. “I have learnt to teach independently and not in collaboration with others.” [I.13].	1. insecure for collaboration 2. used to work individually	1. insecurity 2. unfamiliar with collaboration

## 4 Results

As mentioned above, our research followed the qualitative approach of content analysis. This section presents the results of data analysis in relation to each of the research questions. Τhe answers of the two categories of teachers are presented separately in each research question, in order to allow comparisons between the two groups. As for the first part, of the first research question, the two thematic categories are: (a) negative feelings related to inclusive education, (b) concerns regarding specific SEN students' potential. As for the second part of this research questions the thematic categories are: (a) lack of infrastructure and of differentiated learning material, (b) issues related to the curriculum. Regarding the second research question, the arosen thematic categories are the following: (a) insecurities regarding collaboration, (b) worries for the possibility of deterioration of the quality of teaching, (c) ethics and professionalism of teachers. In respect to the third research question the thematic categories were formed as follows: (a) ‘external sources of resistance to change’, (b) ‘personal sources of resistance to change’.

### 4.1 Guiding beliefs and attitudes towards disability and the feasibility of inclusive practices

The first research question of this study was: What are the guiding beliefs and attitudes of special and general education teachers in respect to disability, as well as to the feasibility of inclusive practices in their daily teaching? Two broader thematic categories were identified in relation to the first part of the first research question concerning general and special teachers’ guiding beliefs and attitudes towards disability, with first, their negative feelings related to inclusive education.

In particular, general education teachers stated that they experience negative feelings, such as fear, insecurities, aversion or ignorance towards disability and, consequently, towards SEN students. As an example, the following was stated: *“As I was teaching, I encountered something unprecedented for me, as I did not expect to see the child's artificial piece of eye on the floor. Nobody prepared me for that, because my colleagues avoided including her in their class”* [I.13].

The negative attitude towards disability derives, and reversely, results in feelings of helplessness: *“nobody told me that this student was diagnosed with bipolar disorder, I didn't know what to do. Fact is that I need instructions”* [I.11]*; “I have a student with mental disabilities in my class. He cannot learn at all. I am in a really difficult situation”* [I.14].

Like their colleagues, special education teachers, stated that they experienced negative feelings towards disability. In respect to the reasons, they suggested at first, their lack of familiarization with disability from the early years of life due to the culture that prevailed in their family environment and which favored the dividing lines concerning these children: *“Our family did not help us, when we were children, to get acquainted with children with this kind of diversity. Disability was something strange to us”* [I.3].

Furthermore, they talked about the negative impact of disability to teachers' psychological condition: *“I would like to help, but not complicated cases. I feel that this has a considerable impact on my psychological state”* [I.2]. This component could be connected, partly, with their anxiety of dealing with cases of severe disability, without having sufficient professional expertise: *“I feel disquiet that I will only have the cases of severe problems or disabilities even in an inclusive setting, because general teachers will claim that they do not have any experience”* [I.6]. *“It seems strange to me to teach in the same class students with severe disabilities and students of typical development. I’ m not ready to teach them all, efficiently”* [I.2].

The second thematic category, regarding the part one of the first research question, was related to concerns referencing specific SEN students' potential. In particular, general education teachers expressed their concerns regarding specific SEN students' potential (e.g., students with severe mental disabilities) to learn effectively in an inclusive class. As reasons, they suggested first of all their doubts regarding students' skill development and progress. Teachers were skeptical regarding SEN students’ potential to learn and develop their skills within this setting: *“Children with mental disabilities cannot learn easily in an inclusive setting. Rather, they have a psychological satisfaction for being with students of typical development”* [I.8]; *“Students with mental disabilities, who take medication, cannot attend successfully this school”* [I.13].

Also, they articulated their concerns regarding SEN students' social participation. Besides their worries about academic achievement, teachers wonder whether SEN students will participate in peer groups and form social relationships with their classmates: *“As this SEN student grows up, students of typical development don't include him in their groups, because they don't have anything in common”* [I.12].

Finally, they expressed their anxiousness of typical students’ reactions to offensive, violent or infringing behavior of SEN students. Teachers described that students with emotional or behavioral problems may be harmful for others or even themselves, creating problems at school: *“My student with special educational needs shows aggressive behavior. He caused an injury and, since then, parents of the other students are not positive about his presence in the class. Also, some students are afraid of him.”* [I.9].

Further, special education teachers also expressed their uncertainties in respect to whether SEN students can successfully attend the mainstream school. As reasons, they stated the severity of SEN. Teachers stated that the severity of the disability is the decisive factor for a student's ability to attend the mainstream classroom and follow the curriculum: *“The truth is that a student with severe disability cannot coexist [with students of typical development], even if a supporter is in the classroom with him. He could just attend the mainstream classroom for 1 or 2 h per day, so that he comes in contact to children without disability. Otherwise, I am wondering, is it really beneficial for him to attend the mainstream classroom?”* [I.2]. *“They don't have enough academic skills. The goal for this child is to develop merely skills for independent living and self-determination”* [I.4].

Also, they highlighted their concerns regarding behavioral problems of SEN students, due to their fatigue, as they are in the classroom for a long time. As a result, they are worried about their impact to the classroom and, consequently, the teachers’ responsibility: *“Teachers prefer children, who do not bother them”* [I.6]; *“If something wrong happens, we are legally accountable”* [I.3].

Regarding teachers’ views on the feasibility of inclusive practices in their teaching, 2 categories were also identified, with first, the lack of infrastructure and of differentiated learning material.

As one of the reasons for their doubts on SEN students’ successful learning in the mainstream classroom (see a.1.2), general education teachers underlined the lack of infrastructure, as well as of differentiated learning material. In fact, it is not uncommon for primary schools not to be equipped with ramps, adequate architectural and spatial adjustments in classrooms, elevators as well as differentiated teaching material and learning supplies for SEN students: *“Books have no differentiated exercises”* [I.9].

Also, special education teachers noted the lack of infrastructure as one of the main barriers that makes schools inaccessible for students with disabilities, which consequently hinders SEN students' to successfully attend the mainstream school. As an example, the following was stated: *“We don't even have a ramp outside the integration class. Did you see the stairs on the front of the entrance?”* [I.5].

In addition, teachers underlined the pressure that they are under for covering the academic school curriculum, which is the second thematic category regarding their views. Specifically, due to the expectations for covering defined areas of the curriculum, teachers feel stressed to achieve goals that concern mainly academic skills within a strict timeframe. Thus, there is not enough time to focus equally on the development of socio-emotional skills, as well as other practical and life relevant abilities that would benefit SEN students.

In respect to that, they note their concerns about their constant stress of being considered as a non-efficient teacher, if the yearly goals of the curriculum are not accomplished: *“Can a SEN student learn to count until* 100 by *the end of the first class of primary school?”* [I.10]; *“We have an academic orientation, because this is expected from us”* [I.15]. Behind the above statements lies the fundamental perception that school should mainly provide academic knowledge: *“Curriculum demands should be less even for students of typical development, much more for SEN students”* [I.10].

Special education teachers expressed their skepticism as to the feasibility of the implementation of the ‘full-inclusion’ model within the current system. In fact, they believe that the general school has a strong academic orientation, without focusing equally on the development of life and social skills, which is beneficial for students with disabilities: *“This student enters the class, but he doesn't earn much, also it's rarer for him compared to his classmates of typical development to make friendships”* [I.2].

Finally, they stated their preference for teaching in special education schools, where they can fully concentrate on the students that they feel responsible for: *“It's very difficult to* support *students with disabilities and students of typical development at the same time”* [I.2]. In the end, they appeared skeptical regarding the feasibility of the ‘full-inclusion’ model, based on the lack of actual examples: *“We speak theoretically. I haven't seen somewhere close inclusive education programs”* [I.1].

### 4.2 Teachers’ definition of their role and of their responsibilities in an inclusive classroom

Both groups of teachers share similar thoughts, as well as concerns, regarding their role and responsibilities in an inclusive classroom. In fact, general schoolteachers point out at their worries and insecurities in respect to collaborative teaching, which comprise the first thematic category related to the second research question. Concretely, they discussed about their sense of ‘ownership’ of the classroom and fear of being challenged, exposed or even having conflicts between colleagues in the context of collaborative teaching. They noted that they don't want any other person to see how they work in the classroom due to the fear of being exposed as not knowledgeable or not competent enough in the eyes of another colleague: *“Teachers don't want anyone to watch the way that they teach. They don't feel comfortable and they make sure to make that clear to their colleagues”* [Ι.12]; *“We cannot collaborate. It will be disastrous. Teaching is an art. It's difficult to share my teaching time”* [I.14].

Subsequently, they talked about their lacking culture for collaboration. Teachers explained that they have neither the training and experience, nor the culture to work as a team. At the same time, they pointed out at the need for common ground and cultivation of a trustful base, in order to build a solid collaboration: *“I have learnt to teach independently and not in collaboration with others. This demands maturity, common way of seeing, a good and trustful relation between partners. We may have a big conflict.”* [I.13].

In addition, they discussed their lack of experience in collaboration. Traditionally, general education teachers are assigned one classroom, holding the entire responsibility for it. Thus, they are used to be the head teacher and are not willing to share it: *“Teachers with over twenty years of experience have their classes ‘closed’, i.e., they are not willing to open it to other colleagues. It is difficult to collaborate”* [I.12].

Besides the above, they referred to the additional time for teaching planning. Collaborative teaching requires enough time for planning and preparation. Taking into account teachers’ work overload and the lack of assigning specific amount of time from their schedule for preparing co-teaching, it is easy to assume that they resist this change that demands more time and effort of them: *“My program would have been much more tiring and overloaded, in order to be prepared enough to teach in a co-teaching class”* [I.8].

General education teachers also shared their worries for the deterioration of teaching and the quality of learning in inclusive classrooms, as part of their responsibility, which consist the second thematic category in respect to the second research question. To that matter, they mentioned the following aspects: Lack of experience a) in planning, b) in time management in co-teaching and, c) in differentiated teaching. The above elements consist new tasks for teachers, who haven't been trained adequately. In addition, they feel responsible for the teaching that they were trained, which - in many cases - doesn't include knowledge on issues of special educational needs: *“It is very hard, we need to be very careful in our planning, students of typical development may have uncommon reactions. I don't have any knowledge in special education planning.”* [I.8]; *“I find it more convenient if SEN students are taught in their special unit, where they follow a specific program, and attend only for few periods the mainstream class*” [I.14].

Special education teachers share similar concerns regarding the collaboration between teachers and the way that they are viewed by their colleagues of general education. First, they mentioned the feeling of ‘ownership’ of the classroom and issues of leadership as one of the most important reasons regarding the difficulties in collaboration. Next to the feeling of being the main responsible for their classroom, teachers tend to be negative towards collaborative teaching, mainly due to issues of ‘ownership’ and leadership within the classroom: *“Who will have the leadership in an inclusive setting with co-teaching?”* [I.3]. *“Often, the special education teacher has more knowledge compared to the general education teacher, so she wants to do more, but feels underestimated by the general teacher, because the latter is used to be the leader in the class”* [I.7].

In addition, they appeared to be more concerned regarding the separation of tasks and roles rather than the collaboration. While co-teaching suggests that the responsibility and the teaching act is mutually shared – in various forms – among the two teachers, however special education teachers often suggest that they are perceived by colleagues as the only ones responsible for SEN students: *“I had a husky student with Autism Spectrum Disorder (henceforth ASD) in crisis and my [general education] colleague was trying to continue with the lesson, leaving me alone to calm the child”* [I.1]. *“We are not familiar with the culture of inclusion”.* [I.3].

Ultimately, they referred to the ethics and professionalism of teachers and that is also the next thematic category that emerges from their answers. They noted that they are confronted with negative views about disability and that their work is not recognized as a contribution that is equal to the other teachers: *“I help and* support *a student with a disability when, for example, he wants to go to the toilet, but the other teachers don't recognize this as a part of the teacher's job. They say, “You have degenerated into a* support *staff”, I have heard it even from my school principal”* [I.7].

### 4.3 Sources of resistance to change towards establishing an inclusive culture and practices in school

The interviews resulted in the accumulation of a series of factors that were suggested as sources of resistance to change towards establishing an inclusive culture and practices in school. The factors were classified as external, referring to issues, such as infrastructure and conditions, and internal, i.e., personal such as fears, insecurities, attitudes or prejudices.

As for the first category, ‘external sources of resistance to change’, general education teachers mentioned first the feeling of routine and work overload. Teachers have already considerable amounts of work, while in specific school periods (such as school fests) they are overloaded, so that they do not wish to add more responsibilities: *“We are used to a specific routine in class, which should be disrupted by the particularities of some students, who are* e.g.*, screaming”*. [I.14]. *“I have to admit that a SEN student needs more time, effort and individualized teaching. Therefore, one would be skeptical to have a student that adds much more work”* [I.13].

Further, the lack of training in inclusive classroom management appeared to be important for them. Teachers stated that they lack experience in handling students, e.g., with behavior problems, in an inclusive setting. Thus, if possible, they stated that they preferred to avoid take over a mixed classroom: *“I don't think it's something I can undertake; I mean to teach two different curricula in the same class”* [I.10].

Regarding special education teachers, they first referred to the continuous rearrangements that are introduced by the Ministry of Education. As they pointed out, over the last decades, it hasn't been an unusual practice for Greek governments to revise or interrupt introduced rearrangements and new educational policies within short periods of time: *“It [i.e., the state] starts a change, but does not fully implement it, the change stops. It is never completed”* [I.7].

Furthermore, same as their colleagues, special education teachers are confronted with work overload – especially in certain periods of the school year –, thus they tend to avoid extra work. In addition, they tend to respond negatively in new ways of work, which might create new challenges. As a consequence, they respond negatively to the option of expanding their workdays: *“We already have too much work to do [at home]”* [I.6]. *“We'll have to be organized from the beginning”* [I.3].

In addition to the above, they face the fear of being challenged by new demands. Teaching in an inclusive class is an unknown situation even for special education teachers, since they are confronted with new demands: *“You will be afraid all the time since you've got an extra role in an inclusive class. You have to be alerted all the time”* [I.6]. “*I'm skeptical about this [teaching in an inclusive class], because if you are quite conscientious, you will want to do your job properly. But what are you supposed to do when something is new and you are not familiar with?”* [I.7] *“[in inclusive settings] It's going to be like we are learning from the beginning our job”* [I.1]. Similar to this, is the lack of sufficient experience and professional expertise in the domain of SEN.

Also important for special education teachers is the fear of parents' reactions. Having referred to the negative reactions of students' parents without disabilities, they underlined the importance of the collaboration with parents as a crucial factor for successful inclusive planning: *“Ι am afraid to make it [teach in an inclusive class]. Students’ (of typical development) parents will be negative as to the possibility of teaching their children together with children with SEN”* [I.4].

‘Personal sources of resistance to change’ is the second category that emerged from the data. Next to the issues that were discussed above (e.g., negative feelings towards disability and SEN students, concerns in respect to collaborative teaching), general education teachers talked about fundamental fears that touch upon their self-awareness and role. For example, one of the biggest concerns of teachers is the fear of degrading their role by taking on supportive tasks without having more significant responsibilities in the classroom responsibility. *“Special schools have knowledge and adequate staff, general schools don't have the same conditions, who is going to* support *these students?”* [I.13].

In addition, they discussed their fears for handling disability that results in feeling challenged to manage a new teaching situation, including SEN students**.** General education teachers stated that they didn't have the expertise to handle effectively the needs of students with disabilities, since their academic studies don't include sufficient training in the field of special and inclusive education. Thus, they felt insecure to organize their teaching appropriately: *“I have experience only in general class, therefore SEN students must be responsibility of the special teacher, because I don't know what to do”* [I.10].

Special education teachers underlined that sometimes they experience feelings of inferiority. Particularly, they noted that and their work is not recognized as equal to their colleagues of general education: *I have felt it even from my school principals' behavior that I'm inferior to my colleagues of general education”* [I.7]. Another teacher suggested that she is perceived by her colleagues as the only one responsible for the SEN students: *“School is unfair -since usually they treat us as inferiors- but we have to accept it since society is also like that.”* [I.3].

Additionally, they face the fear of exposure via collaborative teaching. Similarly, to their general education colleagues, they talked about the fear of being exposed as not skilled enough or not effective enough in the eyes of their colleagues: *“The general education teacher had noticed that the student had serious problems, but didn't say anything, because she was unwilling to collaborate with me. She wanted to look very productive with all the students”* [I.1].

Dealing with a class with more than one student with severe disabilities is also an important fear for them. Even with an expertise in special education, teachers expressed their fear of handling effectively students with severe disabilities: *“I am afraid that I will have only the cases of severe problems or disabilities even in an inclusive setting, because general teachers will claim that they do not have any experience”* [I.6].

At the end, special education teachers, talked about the fear of change. In the context of explaining their reluctance towards initiating, organizing and implementing change, they underlined their fear of change and its implications: *“I am afraid of that kind of changes. We have not sufficiently trained how to teach in inclusive settings”* [I.3].

## 5 Discussion

### 5.1 Summary of results

With this study we aimed at identifying personal sources of resistance to change, as it is suggested by the principles and practices of inclusive education and their impact on teachers' practices. In that respect, we investigated Greek special and general education primary school teachers’ attitudes as well as fears about disability. Our research suggests that both attitudes and fears are related to: first, the school culture and its ethics, second the culture towards disability, third existing views on educational policies and their implementation and, fourth ethics and professionalism of teachers.

In respect to the first research question, i.e., general and special education teachers’ beliefs regarding disability, our data showed that the teachers, who participated in our study, were strongly influenced by the medical paradigm of understanding disability (I1, I2, I 10, I 12, I 13, 14, 15). This becomes apparent, as many of them (I2, I12, I13, I14, I15) expressed feelings of sadness and fear towards disability - especially mental disabilities and behavioral problems. Our findings agree with the work of Lenakaki et al. (2018), who suggest that more complex forms of disability have little acceptance from teachers, who generally prefer to work with students with physical disabilities.

This suggests that 22 years after the implementation of the Greek law 2817/2000 (L. 2817/2000) that ensured the provision of free, quality education for all pupils including students with disabilities, still today one part of Greek educators has limited knowledge in respect to instructing students with disabilities, which leads them to expressing considerable concerns and fears regarding disability. In our study general education teachers viewed disability mainly as the individual student's personal barrier, following the deficit model [35]. In fact, they used frequently the word ‘difficulty’, to describe both the challenges that students with disabilities face and also the challenges (such as uncertainties, fears and stress) that they face themselves, when instructing in classrooms of mixed abilities. Hence, given the fact that the curriculum places an emphasis on academic skills, teachers appear to be skeptical as to the degree that this would suit students with disabilities. Research has shown that a strong academic orientation consists a considerable barrier towards accepting students with disabilities [6,43,57].

On the contrary, special education teachers show stronger readiness and willingness to teach students with disabilities in the mainstream school, as they have an academic background in Special Education. At the same time, part of our sample expressed doubts as to the feasibility of inclusion. In fact, our data suggests that even some of the teachers with an expertise in Special Education are more positive in the perspective of teaching students with specific difficulties, such as with learning disorders and Asperger Syndrome. They expressed fears towards teaching students with complex disabilities, being in favor of separated school settings, where all appropriate equipment and individualized support is provided. That, agree with Ashton, & Arlington (2019) research, were researchers concluded that, prior training of teachers, before their entrance in schools, could reduce their fears toward teaching students of different kind of disabilities [35]. indicated that all teachers appear to be positive towards inclusion, but they emphatically expressed a preference for students with mild disabilities. In addition, they considered students with complex disabilities to be the responsibility of special schools, for the same reasons that our participants suggested. Prior research of [81], also suggested that the education of students with disabilities was considered mainly as the responsibility of special education teachers. Hence, general education teachers are shown to be hesitant towards inclusive education, pointing out at the lack of time, education, expertise and sufficient professional training, findings that are also discussed in the research of [6,45].

Further, some teachers underlined the importance of a personal, prior experience with a person with a disability, pointing out at the impact of prior familiarization with disability, as a factor that enhances acceptance and willingness to work with students with disabilities [6].

Finally, our findings suggest that teachers express unwillingness and concerns regarding change, especially in respect to creating a different school culture and, concretely, one that places an emphasis on stronger collaboration among teachers, differentiation of teaching and implementation of inclusive practices. These findings agree with the theory of fear, because, as we presented earlier at this paper, many of the fears we experience are linked to the culture of fear [31], leading teachers to prefer conditions that offer them safety. Thus, the culture of fear directs teachers to the avoidance of taking any kind of risks by teaching to students of different abilities, as, this is a form of culture that promotes precaution as a virtue and cultivates a climate, where taking risks is even considered irresponsible behavior.

These characteristics/consequences of fear, appeared also to the data that emerged regarding our second research question, were we examined teachers' perceptions of their role and responsibilities in an inclusive classroom. We concluded that while some special education teachers express readiness to differentiate their teaching and collaborate with their colleagues of general education, some others are still hesitant towards experimenting with different forms of teaching. As for general education teachers, they appeared to be unfamiliar with working in an inclusive setting and stated that they are in need of support, as to continue providing good quality in teaching and learning for all students. General education teachers’ lack of expertise and professional training is also discussed in the research work of [80], [45]; and [82]. One of the most interesting findings of our research is that, although teachers of special education are expected to be equipped with efficient training, in order to support the learning process and needs of all students, regardless of the type of special education needs, they appeared to have similar attitudes as teachers of general education regarding the extent and feasibility of inclusive education and culture. This attitude, is also linked to the feeling of fear, as, according to the theory of fear, teachers as individuals is possible to develop resistance to something that causes them anxiety, in spite of the theoretical knowledge they might have [15].

At the same time, general education teachers admit their unwillingness for collaborative teaching in teams with special education teachers, emphasizing strongly the exclusive responsibility over their class. This attitude contributes to creating barriers for successful inclusive practices at school, as it jeopardizes effective teaching in teams of special and general education teachers. This finding agrees with the findings of Lenakaki et al. (2018), research. Research confirms that fears for teachers are: first personal, such as difficulties in collaborative teaching and insecurities regarding handling students with disabilities; second, professional, such as the degradation of their role, by being assigned with supportive tasks rather than significant responsibility for the classroom and, third, class management, such as their confidence in the success of concurrent teaching (Saloviita (2020). Hence, our research points out at teachers’ fears as to co-teaching, together with the challenge of meeting the standards of the academic curriculum and cultivating functional skills as well.

Our last research question concerns teachers’ personal sources of resistance to inclusion and the impact on their teaching effectiveness/practices. Some of the interviewees expressed fears that students with cognitive disabilities have limited capacity to fully participate in the course of the mainstream school. This prejudice builds up on the medical model of understanding disability and shows lack of culture towards disability. Special education teachers, on the other side, are familiar with working one-to-one with students within an integration class, thus it becomes more difficult for them to realize the marginalization and inequity that is reproduced when putting barriers to inclusion, something that emerged from the reserces of [58]; Ampadu, & Suleiman (2018) and [59]. At the same time, general education teachers seem to be less concerned with the morality of these choices, and suggest that different school types are more suitable for students with different needs, something that is also connected to the theory of fear, which has the strength to lead teachers as individuals to prefer the status quo, than taking the risk of working in a different way [15].

Further, all interviewed teachers discuss the work overload that doesn't leave sufficient time for course planning and classroom management, which is necessary in an inclusive setting, evidence also supported by previous research by Salovita (2018).

### 5.2 Theoretical contribution of the research

Thus, our research points out at the lack of substantive collaboration as a fundamental obstacle for implementing inclusive education – finding that agrees with prior research on the topic [[80], [82]]. This weakness is a critical point, as collaboration and working in teams is an important part of the school culture. In fact, school culture defines the school's identity as an organization, as it impacts every member of the school community, their productivity and success in achieving goals. Therefore, our data suggests that lacking: first, shared responsibility over the provision of quality education to all students in the classroom, second, self-confidence of managing classrooms of mixed abilities, third, substantial collaboration between teaching and supportive staff, fourth support in and out of school and, finally, mutual respect of every member's contribution play a significant role in the organization's functioning and in overcoming barriers/difficulties. These findings agree with the work of [13] and [14].

As discussed in the introduction, Senge's theory (2006) describes as a growing institution one that is continually expanding its capacity to create its own future. To achieve this goal, five disciplines must be followed: first, systems thinking (consisting of the interdependence among the organization members), second, collaboration in mental models that promote the establishment of inclusive culture third, commitment to a shared vision, fourth personal mastery and team learning and, fifth, a new definition of leadership.

Through our research, as stated before, we realized that the Greek primary school teachers, who participated in our study, felt comfortable in the status quo of the traditional educational system, that they were familiar with, and showed a certain unwillingness – expressed through fears and resistance to change – to change their practices, according to the theory of fears [31]. Even after a long period of the implementation of integration in Greece as the main educational policy, it's of major importance to realize the need of going a step further as far as the level of practices is concerned [13]. We documented perceptions regarding disability, among teachers that are still guided by the medical model of disability, that have an impact on teachers' professionalism and indicate significant challenges as to the implementation of inclusive principles and practices. As a consequence, the lack of discipline in the principles of inclusive education imply the absence of inclusive school culture, as well as the lack of ‘ethics of inclusive care’, which relates to justice, responsibility and duty and goes beyond any learning difficulty [9]. Our research showed that this is due to a number of factors: first, the notions of disability, second, the lack of adequate training, third, the reluctance for cooperation between teachers, fourth, inadequate infrastructures and curricula and – most importantly – fifth, the lack of inclusive culture (not only among general education, but also among special education teachers). The latter is crucial, as it is linked to a set of values that are fundamental for inclusive education. Since teaching is in its core ethical [60], the teachers' perceptions of disability, the lack of professionalism, as well as ethical professionalism and care, and finally, the lack of strategies of ethical purposes achievement create a negative setting towards disability [48].

In this context, according to Ref. [10] teachers and principals must reconsider the way of approaching their profession. As he elaborates, their attitude towards inclusion originates from their feelings towards social justice in education [10] – finding that strongly emerged in our study. Similar to these were the outcomes of a study that involved Greek-Cypriot teachers [61]. Specifically, his research showed that teachers experience “intense emotional ambivalence in their efforts to cope with growing diversity and multiculturalism in schools” [61]:703).

Given that change evokes strong reactions (Senge, 1997; [62], teachers’ fears are significant factors that hinder the management of change towards inclusion. In fact, teachers seem to feel more comfortable in the safety of their habitual routines. In addition, they expressed skepticism towards top-down inserted changes as well as continually changing educational policies that, in some cases, are not fully implemented, thus considered demanding, superficial and, in the end, not well executed. Such experiences make them frustrated and unwilling to change [15,63]. Therefore, resistance and relying on routine offer them safety [62] and, in consequence, make them negative to top-down suggested change, especially in centralized educational systems [81]. As a result [48], note that there is a lack in ethical demands as to the feeling of “care for the other” (p. 290) that would lead all involved individuals in an ethical collaboration that is, in a collaboration that will promote the fair and equal co-education of students with disabilities, that will promote substantial change towards implementing inclusive practices and culture in Greek primary schools [82].

### 5.3 Limitations

Since we followed a qualitative paradigm of research, the present findings cannot be extended to wider populations. In addition, the research sample is restricted, which however shouldn't be considered as an issue in the context of a qualitative study that sets the focus on participants' deeper thoughts, attitudes and feelings.

### 5.4 Future research

Finally, based on the present qualitative research, we suggest that it would be very important to further investigate the feeling of fear of disability, in relation to issues of collaboration between special and general education teachers and also, the collaboration of teachers with school principals as the leaders of the school community.

## 6 Conclusions

In our study we discussed Greek primary school general and special education teachers' fears towards disability and their impact on teaching students with disabilities in inclusive classrooms. We relied on [38] understanding of schools as learning organizations, which aim at constantly evolving their working models and cultivating collective intelligence for welcoming all students. Our data suggest that the educational system in Greece needs to intensify their efforts towards shifting the existing culture of understanding disability and welcoming diversity in schools. This must be done in different levels: at the level of designing educational policies, at the level of teacher training and at the level of schools. For this purpose, we suggest that schools in Greece could make use of practical educational instruments of self-evaluation and ongoing inclusive development, in order to initiate processes of inclusive self-improvement that take into account the school community as a whole, such as the Index for Inclusion [64]. Finally, a reconsideration of teacher training programs in Greek universities could open the discussion regarding the establishment not only of inclusive principles and practices, but also of ‘inclusive ethics’ as one of the cores of the programs. In the end, the goal is to rethink the importance of discussing the theory and practice of equity and quality in education for all students with the new generations of Greek teachers but also their readiness of coping with new demands on education.

## Production notes

### Author contribution statement

Kyriaki Koullapi: Conceived and designed the experiments; Analyzed and interpreted the data; Contributed reagents, materials, analysis tools or data; Wrote the paper.

Olga Lyra, Assistant Professor, Inclusive Education: Conceived and designed the experiments; Performed the experiments; Analyzed and interpreted the data; Contributed reagents, materials, analysis tools or data; Wrote the paper.

Eirini Kalogeropoulou, Secondary Education Teacher – Master in Special Ed: Conceived and designed the experiments; Performed the experiments; Analyzed and interpreted the data.

### Data availability statement

Data will be made available on request

### Additional information

No additional information is available for this paper.

## Declaration of competing interest

The authors declare that they have no known competing financial interests or personal relationships that could have appeared to influence the work reported in this paper.
